# CsCYT75B1, a Citrus CYTOCHROME P450 Gene, Is Involved in Accumulation of Antioxidant Flavonoids and Induces Drought Tolerance in Transgenic *Arabidopsis*

**DOI:** 10.3390/antiox9020161

**Published:** 2020-02-17

**Authors:** Muhammad Junaid Rao, Yuantao Xu, Xiaomei Tang, Yue Huang, Jihong Liu, Xiuxin Deng, Qiang Xu

**Affiliations:** Key Laboratory of Horticultural Plant Biology (Ministry of Education), Key Laboratory of Biology and Genetic Improvement of Horticultural Crops (Ministry of Agriculture), Huazhong Agricultural University, Wuhan 430070, China; mjunaidrao@webmail.hzau.edu.cn (M.J.R.); xuyuantao@webmail.hzau.edu.cn (Y.X.); tangxiaomeihzau@sina.com (X.T.); yilunhuangyue@webmail.hzau.edu.cn (Y.H.); liujihong@mail.hzau.edu.cn (J.L.); xxdeng@mail.hzau.edu.cn (X.D.)

**Keywords:** citrus, antioxidant flavonoids, drought stress, transgenic *Arabidopsis*, antioxidant enzymatic activity

## Abstract

CYTOCHROME P450s genes are a large gene family in the plant kingdom. Our earlier transcriptome data revealed that a CYTOCHROME P450 gene of *Citrus sinensis* (*CsCYT75B1*) was associated with flavonoid metabolism and was highly induced after drought stress. Here, we characterized the function of *CsCYT75B1* in drought tolerance by overexpressing it in *Arabidopsis thaliana*. Our results demonstrated that the overexpression of the *CsCYT75B1* gene significantly enhanced the total flavonoid contents with increased antioxidant activity in transgenic *Arabidopsis*. The gene expression results showed that several genes that are responsible for the biosynthesis of antioxidant flavonoids were induced by 2–12 fold in transgenic *Arabidopsis* lines. After 14 days of drought stress, all transgenic lines displayed an enhanced tolerance to drought stress along with accumulating antioxidant flavonoids with lower superoxide radicals and reactive oxygen species (ROS) than wild type plants. In addition, drought-stressed transgenic lines possessed higher antioxidant enzymatic activities than wild type transgenic lines. Moreover, the stressed transgenic lines had significantly lower levels of electrolytic leakage than wild type transgenic lines. These results demonstrate that the *CsCYT75B1* gene of sweet orange functions in the metabolism of antioxidant flavonoid and contributes to drought tolerance by elevating ROS scavenging activities.

## 1. Introduction

The economically important and worldwide cultivated citrus fruits possesses abundant amounts of dietary flavonoids, vitamins, minerals, fibers, and folates. Citrus fruits are mostly consumed freshly or as juice (sweet oranges) that becomes a direct source of nutritive metabolites [1]. In a natural environment, citrus plants are exposed to various abiotic stresses such as drought stress. Citrus plants biosynthesize some antioxidant flavonoids to cope against abnormal environmental conditions [2,3,4]. Flavonoid compounds are famous due to their high antioxidant activity that helps to detoxify the free radicals that are produced during drought stress. The flavonoid compounds are further divided into flavanones, flavones, flavanols, anthocyanidins, isoflavones, and flavonols [5]. Citrus is a direct source of antioxidant flavonoids [6] that not only protects plants but also boosts the human immune system and lessens the risk of various chronic diseases such as cancer [7]. Flavonoids possesses significant antioxidant activity in plants as well as in humans, but the biosynthesis mechanism and the genes controlling the flavonoid biosynthesis are less known in citrus [6]. The function of structural pathway genes that are involved in the biosynthesis of flavonoids (which contributes to a high accumulation of antioxidant flavonoids) are the key step to unravel the genetic mechanism behind flavonoids production in citrus [6].

In *Arabidopsis thaliana*, 244 CYTOCHROME P450 (P450s) genes have been identified, and they are mostly involved in various monooxygenation/hydroxylation reactions in secondary metabolic pathways such as the biosynthesis of allelochemicals and flavonoids [8]. P450s genes play a vital role in phytohormones homeostasis and the biosynthesis of different varieties of metabolites. In dicots, some CYP82 (member of CYTOCHROME P450) family genes are involved in protecting plants from unfavorable environmental conditions [9]. The overexpression of the soybean (*Glycine max* L.) cytochrome CYP82A3 gene enhances salinity and drought resistance and has also shown a strong tolerance against biotic stress (*Botrytis cinerea* and *Phytophthora parasitica*) in transgenic *Nicotiana benthamiana* plants [9]. In addition, the rice (*Oryza sativa* L.) DSS1 gene (member of CYTOCHROME P450 gene family) is involved in drought resistance and enhances the growth of rice grains [10]. Some members of P450s genes are involved in the biosynthesis of secondary metabolites, and these genes are actively induced in variety of metabolic pathways [11,12]. Some transcriptomic studies have also shown the regulation of P450s genes during abiotic and biotic stress in cereal [9] and fruit crops [13,14]. However, in citrus species, P450s genes’ functions and mechanisms of regulation have been less studied.

The citrus genome also contains a large number of P450s gene family members [15]. Different transcriptomic studies have shown a significant up-regulation of the P450s gene after biotic stress [13,14]. Moreover, after biotic stress, the citrus plant biosynthesizes some defense-related flavonoid compounds [16]. Interestingly, the flavonoid biosynthesis and up-regulation of P450s genes have been shown to be highly correlated [17]. Many researchers have reported the activation of P450s genes and flavonoid accumulation after various abiotic (e.g., drought and salinity) [11] and biotic (e.g., Huanglongbing—HLB) stresses in citrus [13,14]. Citrus flavonoids have shown a powerful role against abiotic and biotic stresses because of the high antioxidant and antimicrobial activity of their flavonoids, which not only protect plants from reactive oxygen species (ROS) damage but also restrict pathogen (bacteria, fungi, virus, insects, etc.) progression; in addition, these flavonoid compounds are valuable for crop breeding [18,19].

Varied levels of secondary metabolites are present among diverse citrus germplasm, and some have high levels of antioxidant flavonoids such as *Citrus grandis*, *Citrus medica*, and *Poncirus trifoliata* [16], and, interestingly, these citrus species have shown a high expression of P450s genes [13,14]; in addition, these citrus germplasm confer a tolerance against biotic [16] and abiotic stresses [20,21]. However, the biosynthesis mechanism of these antioxidant flavonoids and the regulation of P450s genes are less studied in citrus [6]. The functional annotation of flavonoid pathway genes will be the key step to understand the molecular mechanism that underlies the flavonoid accumulation in citrus [6]. Thus, based on our transcriptome and gene expression, results we have took a *CYTOCHROME P450 75B1* Cs5g11730 gene from *Citrus sinensis* (sweet orange) to overexpress in *Arabidopsis thaliana* (*Arabidopsis*) in order to evaluate its function and possible role under stress. This study not only helps to understand the possible tolerance role of citrus *CYTOCHROME P450 75B1* gene but also assists metabolic engineering to breed a cultivar with increased antioxidant flavonoids in citrus.

## 2. Materials and Methods

### 2.1. Growth Condition and Plant Type

Three independent transgenic lines of *Arabidopsis thaliana* (*Arabidopsis*) were developed for a drought stress experiment. The wild type (WT) *Arabidopsis* seeds (WT ecotype Columbia-0 (Col 0) plants) were sterilized with ethanol 70% (*v/v*) and 100% ethanol for 10 and 8 min, respectively, followed by 4 times washing with autoclaved distilled water. A Murashige and Skoog (MS) medium (with 4.43 g of a MS-dried basal medium (phyto-technology laboratories); 10 g of agar; 25 g of sucrose per liter) was prepared and poured out on the petri plates. The sterilized seeds were placed on the petri-plates with the MS medium and left for ten days in a growth chamber at 20–22 °C. After 10 days, the *Arabidopsis* seedlings were transferred into the soil in culture room for 3 weeks at a temperature about 22 ± 3 °C, 70% relative humidity, and 120 micromoles quanta m^−2^ per sec of light intensity (with a 16/8 h light and dark period).

### 2.2. Agrobacterium Mediated Transformation

The pK7WG2D binary gateway vector was constructed to overexpress the *CsCYT75B1* gene in *Arabidopsis*. The pK7WG2D vector confers kanamycin resistance (because of the neomycin phosphotransferase II (nptII) gene) and also possesses a green fluorescent protein (GFP) (that helps for the manual or visual selection of positive plants) [22]. The *CsCYT75B1* gene was amplified from complementary DNA (cDNA) by using the coding region via PCR, and then the plasmid was extracted. Then the gene was cloned into pDONR221 vector followed by LR clonase (Gateway LR II enzyme) reactions (according to manufacturer’s instructions) and then intervened into a pK7WG2D vector by using gateway technology (Invitrogen). After that, the pK7WG2D was cloned into the GV3101 agrobacterium strain and then transferred into the *Arabidopsis* via the floral dip method [23] to develop transgenic lines.

### 2.3. Transgenic Lines and Drought Stress Conditions

The *Arabidopsis* seeds were sown on the MS medium (with 50 mg/L kanamycin) at each stage for the manual selection of positive plants, and it was later confirmed by DNA extraction (PCR amplification) by CaMV35S (35S promoter) forward and reverse *CsCYT75B1* gene-specific primers. Putative transgenic *Arabidopsis* plants were selected at the T1 generation, and the T4 transgenic lines were prepared by manual and PCR amplification.

For the drought stress experiment, WT, three independent transgenic *Arabidopsis* overexpressed (OX) lines (OX-2, OX-3, OX-5) expressing the *CsCYT75B1* gene were selected, and one empty vector (without the gene) was used. To examine the root, the transgenic plants were exposed to (15%) polyethylene glycol (PEG-6000) osmotic stress for 12 days in a growth chamber, and, after two weeks of stress, the transgenic, wild type, and empty vector plants’ root lengths were checked (with a temperature of about 20 ± 2 °C with 10,000 lux of light intensity). In addition to the soil experiment, the three-week-old *Arabidopsis* plants were subjected to drought stress. The water was stopped after 3 weeks, with following conditions: a 16/8 h light and dark period, 120 micromoles quanta m^−2^ per sec light intensity, a temperature of about 23 ± 2 °C, and 70% relative humidity. The leaf samples were collected at two time points—first, on day 1, and second, after 14 days of drought stress. The leaves were frozen in liquid nitrogen and stored at −80°C for gene expression, metabolic and biochemical analyses.

### 2.4. DNA Extraction and PCR Analysis

The 2% CTAB (hexadecyltrimethyl-ammonium bromide) method was used to extract DNA [24,25], and 0.1–0.2 g (g) of *Arabidopsis* leaves were ground with the help of pestle and mortar followed by the addition of 0.7 milliliters of a DNA buffer (250 mM sodium chloride (NaCl), 200 mM Tris-HCl (pH 7.5), 25 mM ethylene-di-amine-tetra-acetic acid (EDTA), and 0.5% SDS), which was then mixed well, and then left to incubate for 90 min at 65 °C. After incubation, 0.8 milliliters of chloroform–isoamyl alcohol (24:1) was added, and then the mixture was well shaken and centrifuged at 8000 rpm for 10 min; then, the supernatant was collected into a new tube. Then, the DNA pellet was seen in the bottom of tube by adding 1 milliliter of 100% ethanol and 60 microliters of 5 M NaCl [25]. A PCR master mix (Thermo Scientific, Waltham, MA, USA) was used for the amplification of the targeted Cs5g11730 gene according to manufacturer’s instructions.

### 2.5. RNA Isolation and Quantitative PCR

RNA from the fresh leaves of *Arabidopsis* was isolated by using a TRIzol RNA extraction kit (Takara). The total RNA was extracted, and complementary DNA (cDNA) was synthesized as prescribed by producer’s instruction. For cDNA synthesis, 1 μg of the total RNA was used by using HiScript II quantitative real-time (qRT) reverse transcriptase kit along with SuperMix (+gDNA wiper) methodology (Vazyme, R223-01). The cDNA was used for quantitative-polymerase chain reaction (q-PCR) by using the SYBR Green master mix. The q-PCR master mix and all standard procedures were implemented according to manufacturer’s instructions (YEASEN Biotec. Co.Ltd., Shanghai, China). The expression analysis of the pathway genes was conducted by means of q-PCR by using a light cycler 480 II instrument (multi-well plate 384-white; light cycler 480) (Roche). Relative expressions values were determined by using 2^−ΔΔCt^ methodology [26]. For internal reference (control), a β-actin gene from *Arabidopsis* was used. Supplementary Table S1 shows the details of the q-PCR primers used in this study.

### 2.6. Chlorophyll a and b Content

Five hundred milligrams of grounded *Arabidopsis* lead tissues were homogenized in 10 milliliters of 80% (*v*/*v*) acetone [27]. Then, the homogenized mixture was incubated in the dark for 4 h at room temperature (RT), followed by centrifugation for 5 min at 12,000 rpm. For chlorophyll a and b estimation, the supernatant was taken and absorbance was measured at 645 and 663 nm with a spectrophotometer (UV-1800, Shimadzu, Kyoto, Japan), whereas pure acetone was used as blank. Chlorophyll a and b contents were measured in mg/L by means of the following formula:

Chlorophyll a = (OD663 × 12.7) − (OD645 × 2.69) (milligram per liter),

Chlorophyll b = (OD645 × 22.9) − (OD663 × 4.68) (milligram per liter).

In addition, the average fresh and dry weight per plant of the wild type, empty vector and transgenic lines were measured in milligrams (mg) on the first day and after 14 days of drought stress treatment.

### 2.7. Extraction Procedure of Total Flavonoid and Total Phenolis Contents

#### 2.7.1. Extraction

The total flavonoids and total phenolic contents were measured [28]. One-hundred milligrams of leaf tissues were homogenized in 5 milliliters of 80% methanol, followed by 2 h of incubation at room temperature (RT) on an orbital shaker at 200 rpm. Then, after centrifugation at 8000 rpm for 5 min, the supernatant was taken into new tube and this step was repeated for the remaining pellets. Both supernatants were used to determine the total flavonoid and phenolic contents.

#### 2.7.2. Estimation of Total Flavonoid Content (TFCs)

Zero-point-five milliliters of the above-prepared extract was mixed with 2.25 milliliters of distilled water, followed by the addition of 0.15 milliliters of 5% NaNO_2_ (sodium nitrite solution), and, after mixing, it was incubated for at RT for 6 min; 0.3 milliliters of 10% AlCl_3_·6H_2_O (aluminum chloride hexa-hydrate) were mixed well in the above-prepared reaction mixture, followed by 5 min of incubation. After that, 1 milliliter of one molar (M) NaOH (sodium hydroxide) was added and vortexed for 1 min. Then, the total flavonoid content was measured by taking the absorbance at 510 nm on a spectrophotometer (UV-1800, Shimadzu, Kyoto, Japan) [29]. Rutin was used to generate the standard curve, and the total flavonoid contents were expressed in milligrams of rutin equivalents (RE)/gram of dried plant leaf samples (mg RE/g).

#### 2.7.3. Total Phenolic Contents (TPCs)

Total phenolic contents (TPCs) were measured with a Folin–Ciocalteu reagent (FCR), as defined by Velioglu et al. [28]. In a ten milliliter tube, 2.25 milliliters of 10-fold diluted FCR was mixed with 0.3 milliliters of the above-prepared methanolic-extract, followed by 6 min of incubation at RT. After that 2.25 milliliters of the Na_2_CO_3_ (sodium carbonate) (60 g/L) solution was added into the reaction mixture, there was 120 min of incubation at RT. After incubation, the absorbance values were taken at 725 nm on a spectrophotometer (UV-1800, Shimadzu, Kyoto, Japan). Gallic acid (GA) was used to generate the standard curve, and the results were expressed in milligrams of GA-equivalents (GAE)/gram of dehydrated plant leaves (mg GAE/g).

### 2.8. Total Anthocyanin Contents (TACs)

For total anthocyanin contents (TAC) determination, 0.1 g of leaf tissues were homogenized in five volumes (by sample weight) of the reaction mixture (containing 5% acetic acid (*v/v*) and 45% methanol *v*/*v*), followed by centrifugation for 10 min at 10,000 rpm at RT, as described in [30,31]. The absorbance was taken at 530 and 657 nm on a spectrophotometer (UV-1800, Shimadzu, Kyoto, Japan), and the total anthocyanin contents were measured by using the following formula, which eliminated the chlorophylls values:

TAC (mg/100g of dried weight) = (absorbance at 530 nm − (0.25 × absorbance at 657 nm) × 5 times extraction volume (milliliter) × 1/weight of leaf tissue sample (g).

### 2.9. Antioxidant Enzymatic Activity

For the antioxidant enzyme assay, the superoxide dismutase (SOD), catalase (CAT) and peroxidase (POD) activity were measured by using Nanjing Jiancheng bioengineering institute kit. For SOD, CAT and POD activity, A001-1-1, A007-1-1, and A084-3-1 kits were purchased, respectively. The total protein contents were also measured by using an A045-2-2 protein extraction kit from Nanjing Jiancheng bioengineering institute. Five-hundred milligrams of leaves were crushed into powder in liquid nitrogen, and the following procedure was conducted, according to manufacturer’s instructions for SOD, CAT, POD and protein determination.

### 2.10. Malondialdehyde and Electrolytic Leakage

The malondialdehyde (MDA) contents were measured by using Nanjing Jiancheng A003-1 bioengineering institute kit. One-hundred milligrams of leaves ere homogenized according to the producer’s instructions.

For electrolytic leakage measurement, an Ohaus Company Starter3100C apparatus was used. At first, the leaves were cut into small pieces, and then leaf samples were left in deionized water for 20 min in a 200 rpm shaker at room temperature; finally, the electrolytic leakage was measured. After that, the sample solution was boiled for 15 min and then checked again with a Starter3100C apparatus. The electrolytic leakage percentage was calculated according to the formula described in [32].

### 2.11. Hydrogen Peroxide (H_2_O_2_)

Zero-point-one milligrams of leaf tissues were homogenized in 1 milliliter of 1% trichloro-acetic acid in an ice bath, followed by 10 min of centrifugation at 10,000 rpm. Then, 0.5 milliliter of supernatant was mixed with 0.5 milliliters of a potassium phosphate buffer (10 millimolar) in a new tube followed by the addition of 1 milliliter of 1 M KI (potassium iodide). Then, for hydrogen peroxide determination, the absorbance of the reaction mixture was taken at 390 nm on a spectrophotometer (UV-1800, Shimadzu, Kyoto, Japan). Commercial H_2_O_2_ was used to generate the standard curve, and the H_2_O_2_ values were represented by micromoles/g of dehydrated samples [33].

### 2.12. Reactive Oxygen Species and Superoxide Radicals Determination

The reactive oxygen species were determined by using kit (Elabscience: CAT No: E-BC-K138-F), and ROS were calculated according to manufacturer’s instructions with little modifications. The superoxide radicals (O_2_^−^) were measured in 0.1 g of fresh leaf tissues [34]. The one unit of superoxide radical was defined as 0.1 units of change in absorbance per min at the corresponding wavelength values.

### 2.13. Antioxidant Activity and Capacity (DPPH Free Radical Scavenging Assay)

For antioxidant activity and capacity, 0.1 g of leaf tissues were grounded into fine powder, followed by the addition of 1 milliliter of an extraction solution that contained 70% ethanol, 29% water, and 1% acetic acid. After being centrifuged at 8000 rpm, the 0.03 milliliters of supernatant were taken into a fresh tube, followed by the addition of 2.97 milliliters of 2,2-diphenyl-1-picrylhydrazyl (DPPH 0.1 millimolar) and incubated in the dark at RT for 30 min. Then, the absorbance readings were taken on a spectrophotometer (UV-1800, Shimadzu, Kyoto, Japan) at 517 nm. For control, 0.03 milliliters of extraction solution was added to 2.97 milliliters of DPPH [35]. For antioxidant (free radical scavenging) activity, the following formula was used:

Antioxidant activity (%) = [1 − {sample OD/control OD}] × 100.

For antioxidant capacity, Trolox was used to generate the standard curve, and samples values were represented in (millimolar of Trolox/100 milligram).

### 2.14. Trypan Blue and Nitro-Blue Tetrazolium Staining

For the trypan blue staining, *Arabidopsis* leaves were dipped for half hour in the trypan blue staining liquid. Then, the liquid was poured-out, and the leaves were boiled in 96% ethanol for 8 min, as determined by Daudi et al. [36]. For histo-chemical (superoxide) determination, one leaf was taken and stained for 30 min in the nitro-blue tetrazolium (NBT) solution; then, the leaves were decolorized by boiling them in 96% ethanol for 10 min [37].

### 2.15. Statistical Analysis

All the data represented in this study were statistically analyzed by using Statistix 8.1 (Tallahassee, FL, USA) statistical software. The graphs and standard error were determined by using the Excel (Microsoft Corp., Redmond, WA, USA) program. Differences were measured significant at *p* < 0.05.

## 3. Results

### 3.1. Transcriptome and Gene Expression of CsCYT75B1 Gene

The citrus species contains huge variations and dissimilar levels of antioxidant metabolites; some citrus germplasm possess a high level of antioxidant flavonoids, and some have lower levels [21]. Previous transcriptomic studies on citrus metabolites revealed that several CYTOCHROME P450 genes were differentially expressed in different species of citrus [13,14]. Thus, we analyzed our transcriptome data (Supplementary File S2) corresponding to the metabolic data (Supplementary Figure S1 and Table S2) which suggested that *CYTOCHROME P450 75B1* gene expression pattern was correlated (Supplementary Figure S2) with metabolic data in *Citrus sinensis* (Supplementary Figure S1). In addition, we exposed the sweet orange seedlings to different abiotic stresses such as drought stress and high light stress. Under drought stress, the *CsCYT75B1* gene was significantly expressed (Supplementary Figure S3; citrus primers Table S3). Thus, we cloned the *CsCYT75B1* (ID: Cs5g11730) gene from *Citrus sinensis* (sweet orange) to overexpress in *Arabidopsis thaliana* (*Arabidopsis*) to evaluate its function and possible role in stress.

### 3.2. Phylogenetic Analysis of CsCYT75B1 Gene

**Figure 1:**
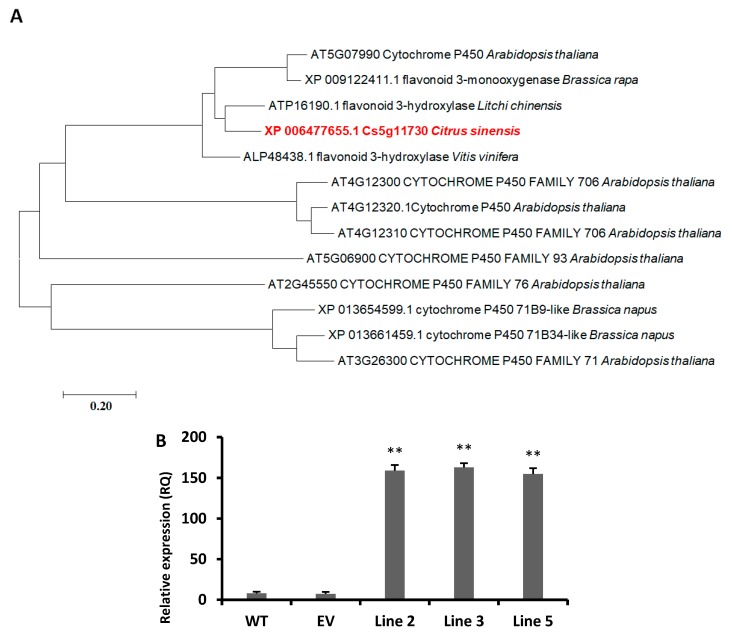
Phylogenetic analysis and gene expression pattern of the *CsCYT75B1* (CYT) gene. (**A**) Phylogenetic analysis of *CsCYT75B1* gene with its homologous genes from other plant species. (**B**) Gene expression pattern of *CsCYT75B1* in overexpressed (OX) *Arabidopsis* lines. Wild type (WT), and empty vector (EV) plants. A Students *t*-test was used to compare CYT-OX and WT at ** *p* < 0.01. Each value is a mean of three replicates.

The protein sequence of *CsCYT75B1* was taken from the citrus genome initiative website (http://citrus.hzau.edu.cn/orange/download/index.php), and we did protein BLAST (Basic Local Alignment Search Tool) in TAIR (The Arabidopsis Information Resource) (https://www.arabidopsis.org/) and NCBI (National Center for Biotechnology Information) (https://www.ncbi.nlm.nih.gov/), and the results are summarized (Figure 1A). The *CsCYT75B1* protein was similar to the *Arabidopsis CYTOCHROME P450 75B1* gene with the highest protein homology (Figure 1). In addition, the *CsCYT75B1* protein homology was aligned with *Litchi chinensis* and *Vitis vinifera* genes that encoded the flavonoid 3-hydroxylase enzyme whereas, in *Arabidopsis thaliana,* the homologous gene of *CsCYT75B1* was responsible for flavonoid 3-monooxygenase (ID: AT5G07990) activities (Figure 1). This AT5G07990 gene is involved in flavonol biosynthesis, leucopelargonidin, leucodelphinidin, leucocyanidin and luteolin biosynthesis in *Arabidopsis thaliana* [38,39]. Thus, phylogenetic analysis showed that the citrus *CsCYT75B1* gene probably had a similar function, as discussed above (flavonoids 3-hydroxylase or flavonoid 3-monooxygenase).

### 3.3. Root Elongation in CYT75B1 Transgenic Lines

**Figure 2:**
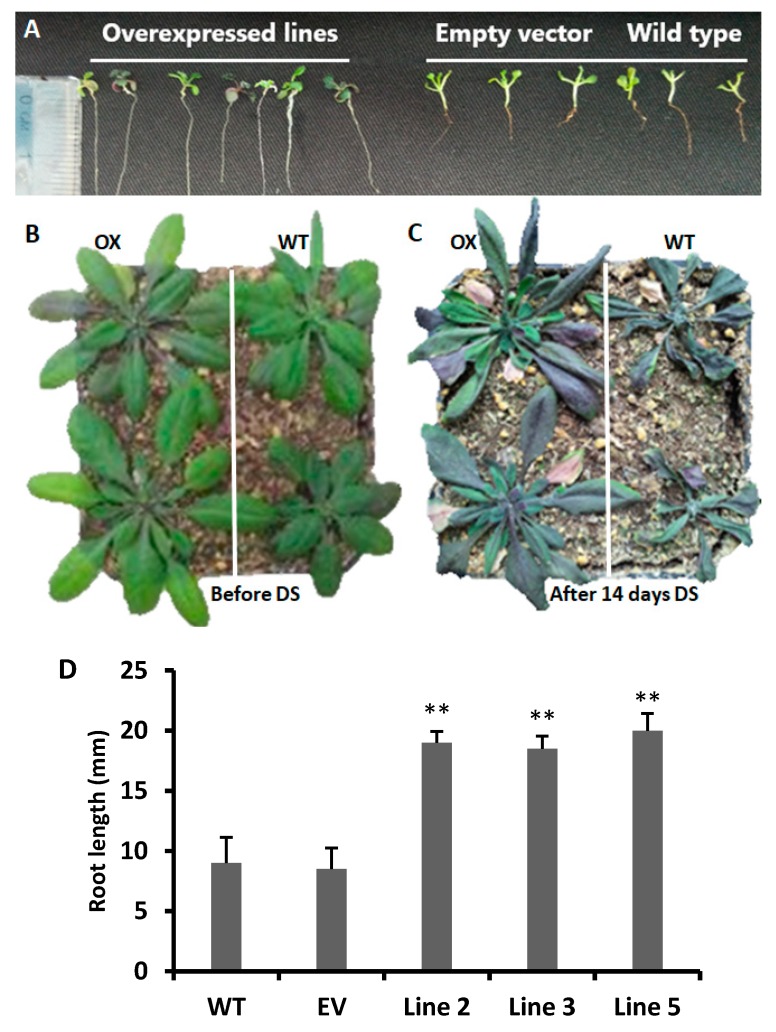
Drought stress effects on transgenic and wild type plants. (**A**) Roots of overexpressed lines, empty vector, and wild type plants after 14 days of polyethylene glycol (PEG-6000) stress. (**B**) Before drought stress (DS), overexpressed (OX), and wild type (WT) plants. (**C**) OX and WT plants after 14 days of drought stress. (**D**) Root length of WT and empty vector (EV) plants compared to the transgenic lines under PEG stress. A Students *t*-test was used to compare the CYT-OX and WT plants at ** *p* < 0.01. Each value is a mean of three replicates.

In the T1 generation, on the basis of gene expression analysis, we selected three independent transgenic lines of *CYTOCHROME P450 75B1* (Lines 2, 3, and 5) gene. For a further drought stress experiment, the T4 generation was obtained, and we grew the transgenic, EV and wild type *Arabidopsis* seeds under PEG-6000 stress. After two weeks, the root length was measured with a ruler in millimeters (Figure 2A,D). The root length was significantly higher in all transgenic lines than the empty vector and wild type roots (Figure 2D). In addition, a few plants showed a purple color phenotype in the leaves after two weeks of PEG-6000 stress (Figure 2A). The overexpression of the *SoCYP85A1* (from *Spinach oleracea*) gene enhances root elongation and confers drought tolerance in transgenic tobacco plants [40]. Moreover, for the soil drought stress experiment, nine plants were randomly selected, and three-week-old plants were subjected to drought stress (Figure 2B). In both the PEG-6000 and soil drought stress experiments, the gene expression results showed that the *CsCYT75B1* gene was up-regulated more than 20-fold in all transgenic lines, as compared to the wild type line (Figure 1B).

The three-week-old transgenic and wild type plants (Figure 2B) were exposed to 14 days of drought stress, and they showed different types of visual symptoms (Figure 2C). Up to eight days of drought stress, the visual symptoms of the transgenic and wild type plants were the same, but, after that, the wild type plants showed some wilting, chlorosis (yellowing), and drying symptoms due to drought stress; similar symptoms were observed in the empty vector leaves, whereas the transgenic plants showed a dark purple color phenotype in their leaves (Figure 2C). In addition, the average fresh weight (per plant) of three-week-old wild type, empty vector and overexpressed *Arabidopsis* lines before treatment was 706, 697 and 702.5 mg, respectively, whereas the average dry weight (per plant) was 67.5, 65.25 and 66 mg, respectively. After 14 days of drought stress treatment, the average fresh weight (per plant) was 203, 198 and 311.5 mg, while the average dry weight (per plant) was 40, 38.5 and 53 mg in the WT, empty vector and overexpressed lines, respectively.

### 3.4. Increased Antioxidant Flavonoids in Transgenic Arabidopsis Lines

**Figure 3:**
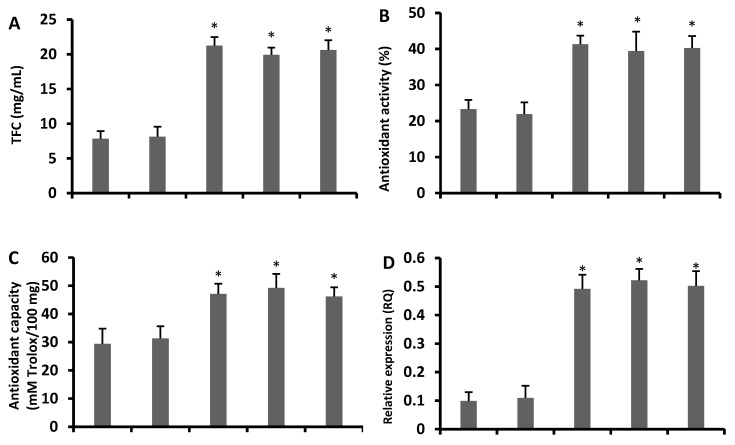

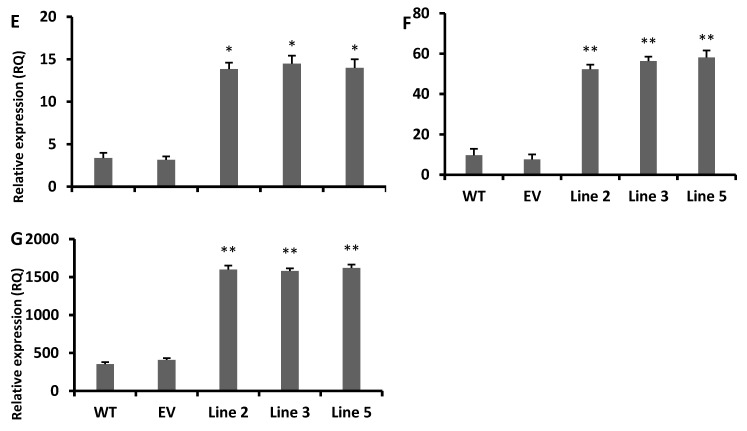
The gene expressions, total flavonoid contents and antioxidant activities of wild type and overexpressed lines without stress: (**A**) Total flavonoid contents, (**B**) antioxidant activity, (**C**) antioxidant capacity, (**D**) AT5G08640 flavonol synthase (FLS), (**E**) AT5G13930 TT4, (**F**) AT3G55120 TT5, and (**G**) AT3G51240 TT6. (WT) wild type and (EV) empty vector. A Student’s *t*-test was used to compare the CYT-OX and WT plants at * *p* < 0.05 and ** *p* < 0.01. Each value is a mean of three replicates.

A higher concentration of total flavonoid contents than that of the wild type plants was observed in all transgenic lines (Figure 3A). In addition, fourteen genes related to flavonoid and anthocyanin biosynthesis were studied in the *CsCYT75B1* transgenic *Arabidopsis* lines. Out of them, some flavonoid biosynthesis genes such as AT5G13930 (TT4), AT3G55120 (TT5), AT3G51240 (TT6) and AT5G08640 (flavonol synthase—FLS) were more than four-fold induced in all transgenic lines (Figure 3D–G), whereas several other flavonoid biosynthesis pathway genes were two- or less than two-fold up-regulated in all transgenic lines compared to the wild type (Supplementary Figure S4). Thus, the high expression of genes related to the biosynthesis of antioxidant flavonoids and the increased level of total flavonoid contents indicated that overexpression of the *CsCYT75B1* gene was correlated with the accumulation of antioxidant flavonoids in the leaves of transgenic plants, rather than the wild type plants.

In addition, the antioxidant activity and capacity of all transgenic lines were considerably higher than the empty vector and wild type plants, which showed that the transgenic lines had a stronger free radical scavenging capability than the wild type plants (Figure 3B,C). Generally, TT4, TT5 and TT6 genes are involved in the biosynthesis of antioxidant flavonoids such as flavones and flavonols [12], and their antioxidant activity can be measured by a DPPH test [41]. Here, the DPPH test revealed a higher antioxidant activity, probably due to the high accumulation of antioxidant flavonoids (Figure 3A–C).

### 3.5. Accumulation of Antioxidant Flavonoids in Overexpressed Lines after Drought Stress

**Figure 4:**
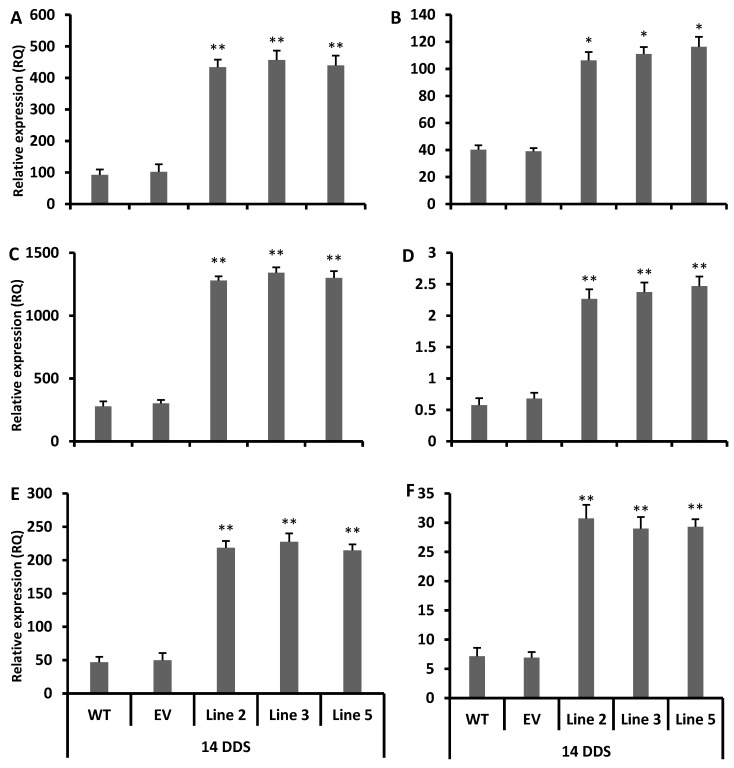
The q-PCR relative expression data of 6 genes under drought stress: (**A**) AT5G13930 TT4, (**B**) AT3G55120 TT5, (**C**) AT3G51240 TT6, (**D**) AT5G08640 FLS, (**E**) AT3G13610 F6′H1, and (**F**) AT4G22880 TT18. Gene IDs were taken from *Arabidopsis* genome website TAIR (https://www.arabidopsis.org/). FLS: flavonol synthase. 14DDS: after 14 days of drought stress. WT: wild type. EV: empty vector. A Students *t*-test was used to compare the CYT-OX and WT plants at * *p* < 0.05 and ** *p* < 0.01. Each value is a mean of three replicates.

The gene expression analysis revealed that the TT4, TT5, TT6, FLS, AT4G22880 (TT18) and AT3G13610 (F6′H1) genes were significantly induced (more than four-fold) after drought stress (Figure 4A–F), while some genes such as AT4G12334, AT5G42800 (TT3), AT4G09820 (TT8) and AT3G28430 (TT9) were more up-regulated more than two-to-four fold in all drought-stressed transgenic *Arabidopsis* lines than in the wild type plants (Supplementary Figure S1.4). In addition, some genes were less than two-fold up-regulated, including AT1G17260 (TT13), and some were down-regulated, including AT1G61720 BAN (BANYULS; negative regulator of flavonoid biosynthesis) and AT3G55970 JOX3 (JASMONIC ACID OXIDASE 3) in all transgenic lines, as compared to the wild type plants (Supplementary Figure S4).

**Figure 5:**
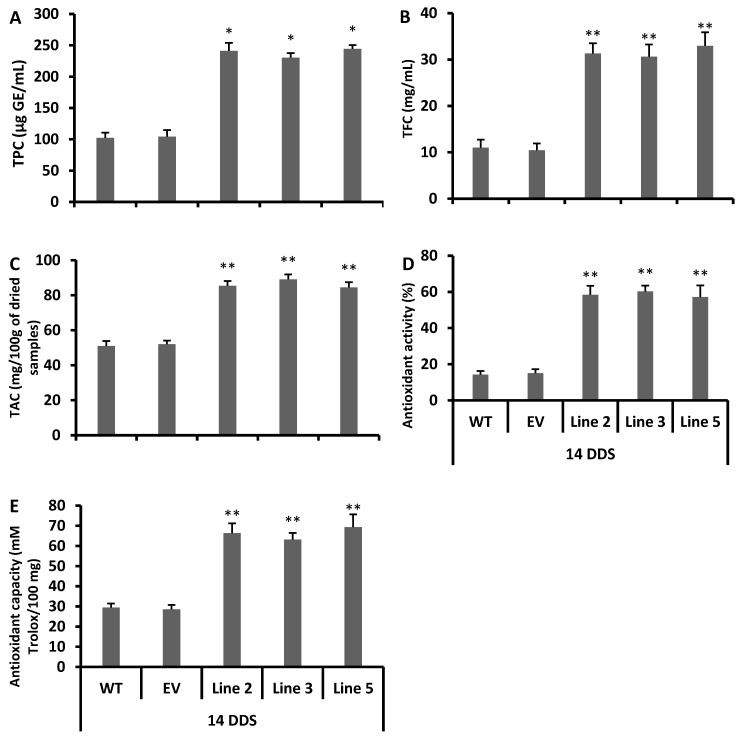
Various metabolic and antioxidant-related parameters. (**A**) Total phenolic contents (TPCs). (**B**) Total flavonoid contents (TFCs), (**C**) Total anthocyanin contents (TACs). (**D**) Antioxidant activity (**E**) Antioxidant capacity. 14DDS: after 14 days of drought stress. WT: wild type. EV: empty vector. A Students *t*-test was used to compare the CYT-OX and WT plants at * *p* < 0.05 and ** *p* < 0.01. Each value is a mean of three replicates.

The total contents of flavonoids, phenolic and anthocyanins were also higher in all drought-stressed transgenic lines than the empty vector lines and wild type plants (Figure 5A–C). In addition, the stressed transgenic lines possessed a stronger antioxidant activity and capacity (Figure 5D,E) than the wild type plants. To conclude the phenotypic analysis, gene expression and metabolic data suggested that the transgenic lines strongly acclimatized the drought stress via the rapid accumulation of antioxidant flavonoids, as compared to the empty vector and wild type plants.

**Figure 6:**
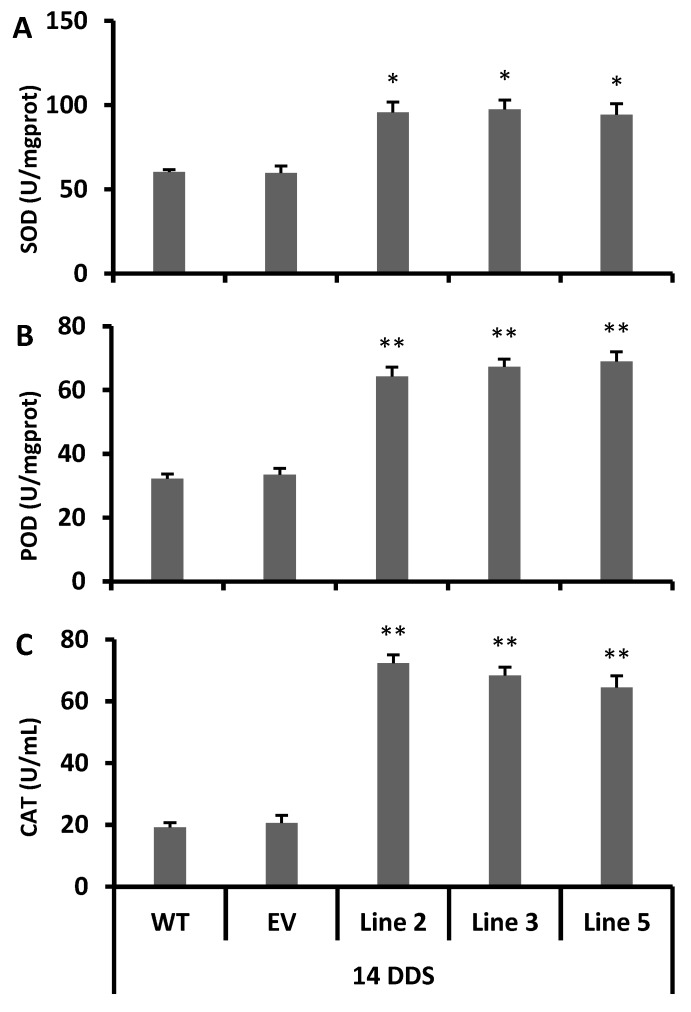
Antioxidant enzymatic activities under drought stress. (**A**) Superoxide dismutase (SOD), (**B**) Peroxidase (POD), and (**C**) Catalase (CAT). 14DDS: after 14 days of drought stress. WT: wild type. EV: empty vector. A Students *t*-test was used to compare the CYT-OX and WT plants at * *p* < 0.05 and ** *p* < 0.01. Each value is a mean of three replicates.

**Figure 7:**
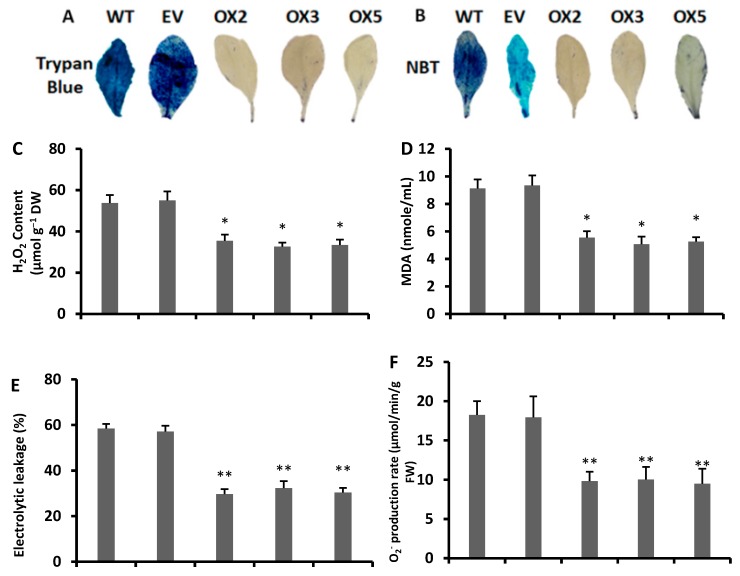

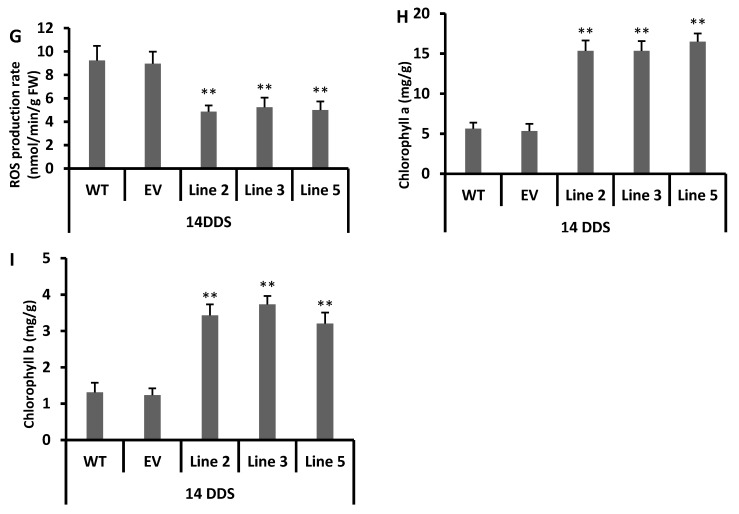
Drought stress effects on transgenic and wild type plants. (**A**) Trypan blue staining effect on OX and WT plants. (**B**) Nitro-blue tetrazolium (NBT) staining effect on OX and WT plants. (**C**) Hydrogen peroxide (H_2_O_2_ content). (**D**) Malondialdehyde (MDA). (**E**) Electrolytic leakage (EL). (**F**) Superoxide radicals production rate (O_2_^−^). (**G**) Reactive oxygen species (ROS). (**H**) Chlorophyll a (**I**) Chlorophyll b. 14DDS: after 14 days of drought stress. WT: wild type. EV: empty vector. OX: overexpressed lines. A Students *t*-test was used to compare the CYT-OX and WT plants at * *p* < 0.05 and ** *p* < 0.01. Each value is a mean of three replicates.

To summarize, 10 genes were more significantly induced after 14 days of drought stress in transgenic lines than in the wild type plants, and these genes were related to the antioxidant flavonoids and the anthocyanin biosynthesis pathway (Figure 4A–F). Usually, the TT4, TT5, TT6, TT18 and F6′H1 genes are involved in the biosynthesis of flavones (e.g., luteolin) its derivatives and flavonols (e.g., quercetin), and these two sub-classes of flavonoids have resilient antioxidant activities [12,41,42,43,44,45]. Our DPPH free radical scavenging results showed that all transgenic lines had stronger antioxidant activity after 14 days of drought stress than the empty vector lines and wild type plants (Figure 5D,E). Thus, our study showed a significant induction of the TT4, TT5, TT6 and FLS genes (Figure 3D–G and Figure 4A–D) with a higher total contents of flavonoids (Figure 3A and Figure 5B), which clearly indicates that the *CsCYT75B1* gene stimulated the biosynthesis of antioxidant flavonoids, which strongly scavenged the free radicals that were produced during drought stress and contributed to drought stress tolerance. Significantly higher antioxidant enzymatic activity was observed in all drought stress transgenic lines than in the WT plants (Figure 6A–C). The dramatic increment in total antioxidant activity and capacity (Figure 5D,E) with higher SOD, POD and CAT activities strongly indicated that the overexpressed lines possessed a more resilient free radical scavenging potential than those of the wild type plants. In addition, there were significantly higher levels superoxide radicals and reactive oxygen species in the wild type and empty vector lines than in the transgenic lines (Figure 7F,G). In conclusion, the wild type plants were more prone to superoxide radicals and ROS damage during drought stress. Drought-tolerant citrus varieties showed significantly higher antioxidant enzymatic activities and antioxidant capacity [20,45,46,47] than the wild type plants; a significant increment in antioxidant enzymatic activities is correlated with drought tolerance in citrus [20]. Thus, our results demonstrated that the overexpression of the *CsCYT75B1* gene contributes to drought tolerance via the high accumulation of antioxidant flavonoids with better antioxidant enzymatic activities (free radical scavenging capability) in all transgenic *Arabidopsis* lines.

### 3.6. Decreased Level of Reactive Oxygen Species in Transgenic Arabidopsis under Drought Stress

The trypan blue staining showed that large areas of the leaves (becomes blue) were more damaged by drought stress in the wild type and empty vector lines than in the transgenic leaves (Figure 7A). The NBT staining showed a high accumulation of superoxide radicals in the wild type and empty vector plants (shown in the blue color) compared to the transgenic plants (Figure 7B). In addition, the superoxide radicals (O_2_^−^) and ROS results showed that the wild type and empty vector lines had significantly higher level of free radical O_2_^−^ and ROS than the transgenic lines (Figure 7F,G). These results confirmed that the wild type and empty vector lines were more damaged and under higher drought stress than the transgenic *Arabidopsis* lines.

The contents of hydrogen peroxide, MDA, and electrolytic leakage were significantly reduced after 14 days of drought stress (14DDS) in all transgenic lines, as compared to the WT plants (Figure 7C–E), whereas after 14DDS, all the transgenic lines possessed significantly higher levels of chlorophyll a and b in leaves than the WT plants. Moreover, our results showed that transgenic lines had higher chlorophyll a and b levels (Figure 7H,I), with a significantly lower concentration of electrolytic leakage, H_2_O_2_ and MDA in all drought-stressed transgenic lines than those of the WT and empty vector lines; these results visibly indicated that the transgenic lines have more potential to tolerate the drought stress, while the wild type plants are more prone to drought stress due to H_2_O_2_-mediated cellular damage.

## 4. Discussion

In plants, the CYTOCHROME P450s gene family has crucial role in the biosynthesis of antioxidant metabolites, diversity, and regulation [48]. In *Citrus sinensis*, a total number of 202 CYTOCHROME P450 genes have been identified [49]. These CYTOCHROME P450 genes have a huge variability across the citrus genera [49]. These variability’s and the complex classification of CYTOCHROME P450 genes leads to diverse functions in plants [50]. Usually, these genes are involved in secondary metabolite biosynthesis [50] and defense responses against various abiotic and biotic stresses [50,51]. Plant CYTOCHROME genes activate during stress conditions and accumulate antioxidant flavonoids [12] to alleviate the negative effects of environmental stress [52].

The *CYTOCHROME P450 75B1* gene is positively correlated with flavonoids [39] flavones and antioxidant flavonol accumulation in *Arabidopsis thaliana* [8]. Flavone biosynthesis is governed by the *CYTOCHROME P450 75B4* gene in rice [17]. To the authors’ knowledge, CYTOCHROME P450 genes function have been less studied in citrus; however, *CYTOCHROME P450 75B1* homologous genes in different citrus species were up-regulated in some transcriptional studies conducted on HLB-infected citrus [13,14]. Our phylogenetic analysis revealed that *CsCYT75B1* had homology with the *Litchi chinensis* and *Vitis vinifera* flavonoid 3-hydroxylase gene and in *Arabidopsis thaliana* with the *CYTOCHROME P450 75B1* gene (Figure 1A). Based on these results, we can predict that citrus *CsCYT75B1* probably has a similar role in citrus, as in other plants.

In citrus under drought stress, many flavonoids such as rutin, rhoifolin and vicenin are significantly induced in leaves, while in roots, some phenolics such as prenylated coumarins are in high concentration [53]. These flavonoid compounds are considered to be non-enzymatic antioxidants that mitigate the negative effect of ROS in plants [54]. Plants alter their flavonoid biosynthesis to cope against unfavorable environmental conditions [55]; in addition, the plants who maintain high concentration of flavonoids during oxidative stress better-tolerate the drought stress, probably due to the high antioxidant potential of flavonoids [56]. Our gene expression results showed that up-regulation of flavonoid pathway genes (Figure 3D–G) with increased levels of total flavonoid contents (Figure 3A) and higher antioxidant activity (Figure 3B) in all transgenic lines (without stress) than in the wild type plants, which clearly indicates that the overexpression of the citrus *CsCYT75B1* gene enhances the antioxidant flavonoid contents in the leaves of transgenic *Arabidopsis*. In addition, after 14 days of drought stress, flavonoid and anthocyanin pathway genes (Figure 4A–F) were significantly induced in transgenic lines, and all transgenic lines more-significantly accumulated phenolics, flavonoids and anthocyanin contents (Figure 5A–C) than the wild type and empty vector lines.

A high accumulation of antioxidant flavonols and flavones might be beneficial for citrus to mitigate the injurious effects of ROS species during abiotic stress [11]. The total phenolic contents were significantly increased when drought stress was imposed on *Solanum scabrum* and *Solanum scabrum* Mill [57]. Several plant phenolic compounds such as derivatives of phenylpropanoids and some amino acids also possess significant antioxidant activity and help plants to acclimatize in stressed environmental situations [58]. Here, our results indicated that the all transgenic lines possessed a higher total phenolic contents than the wild type plants, and similar results were observed after 14 days of drought stress (Figure 5A). According to our gene expression results, the citrus *CsCYT75B1* gene is linked with the biosynthesis of antioxidant flavonoids (Figure 3D–G) that have a higher antioxidant activity and capacity (Figure 3B,C).

Anthocyanin compounds also possess a strong antioxidant potential and help plants to minimize the ROS damage [59]. Since anthocyanins are end products of flavonoid biosynthesis pathways, a high accumulation of flavonoids before stress (due to *CsCYT74B1*) probably aids the rapid biosynthesis of anthocyanin after drought stress. In addition, many plant flavonoids, anthocyanins and phenolic compounds are famous due to their strong antioxidant activities (free radical scavenging) during stress condition [57]. The phenolic compounds such as flavonoids and anthocyanins protect plant cells from the oxidative damage caused by drought stress [12,57]. Thus, our gene expression results showed a high accumulation of antioxidant flavonoid and anthocyanin contents (after more drought stress in all transgenic lines than in the wild type plants) that might be helpful to alleviate the negative effect of drought stress.

The high expression of the TT4, TT5, TT6 and FLS genes in *Arabidopsis* facilitates the biosynthesis of antioxidant flavonoids that contribute to drought tolerance [11,12]. In addition, the TT8 gene is a regulatory factor that stimulates many flavonoid biosynthesis pathway genes and facilitates the accumulation of more antioxidant flavonoid compounds in plants [60]. This gene is also involved in anthocyanin production through the regulation of JAZ (JASMONATE-ZIM DOMAIN) proteins. The BAN gene is a negative regulator of flavonoid biosynthesis [60]. Our results showed that TT8 was up-regulated, while the BAN gene was down-regulated in all transgenic lines after drought stress. In addition, several flavonoid biosynthesis pathway genes such as TT3, TT4, TT5, TT6, TT7, TT8, TT9, TT18, FLS and F6′H1 (involved in the biosynthesis of flavanone, flavone, flavonol, anthocyanin and its derivatives [38]) were more up-regulated after drought stress in all transgenic lines than in the wild type plants (Figure 4A–F, Supplementary Figure S4). The up-regulation of these genes triggers the accumulation of flavonoids in *Arabidopsis* leaves [39]. These subclasses of flavonoids (flavanone, flavone, flavonol, anthocyanin, and its derivatives) possess significant antioxidant and antimicrobial activities [1]; additionally, several compounds related to these flavonoids subclasses have been found to be accumulated in different plant species in response to abiotic stress [12,61,62] and biotic stress [16], and they have been shown to possess strong antioxidant activities and to contribute to drought tolerance [11,12].

Among citrus rootstocks, the genotype that maintain highest antioxidant enzymatic activity (SOD, POD, and CAT) with a higher total antioxidant activity and capacity during drought stress are considered to be drought-tolerant, and the genotype that possesses the lower antioxidant enzymatic activity is considered to be drought-sensitive—that genotype has shown serve drought symptoms on leaves [20]. The total antioxidant activity and capacity increases significantly after drought stress in *Solanum scabrum* Mill and *Solanum scabrum* [57]. Keeping high antioxidant capacity and antioxidant enzymatic activity during drought stress can assist crop plants to acclimatize or withstand stress conditions by quenching the ROS that are generated during oxidative stress, thus contributing to drought tolerance, while lower or decreased in antioxidant activity after drought stress causes the high accumulation of ROS, which disrupts the redox balance and causes drought sensitivity and serve oxidative (oxidation) damage to cellular components [63]. Furthermore, prolong drought stress in different citrus rootstocks causes a dramatic increase in antioxidant enzymatic activities (SOD, POD, and CAT) and contributes to drought tolerance. According to our results, the *CsCYT75B1* transgenic lines showed a significant increment in antioxidant enzymatic activities (Figure 6A–C) with higher total antioxidant activity (Figure 5D) and capacity (Figure 5E) after 14 days of drought stress than the wild type plants. In addition, the transgenic lines had lower superoxide radicals (Figure 7F) and ROS (Figure 7G) than the wild type plants, which clearly indicates that the transgenic *Arabidopsis* lines had less free radicals and ROS, as well as a better antioxidant enzymatic defense system than the wild type plants.

Drought stress has been shown to confer a significant degradation of photosynthetic pigments such as chlorophyll a and b contents in the leaves of *Amaranthus tricolor,* with a reduced overall efficacy of the photosynthetic apparatus [64]. In another study, the same results were observed in different citrus rootstocks; chlorophyll a and b degraded as the drought stress was prolonged in the leaves of various citrus rootstocks [20]. Correspondingly, other abiotic stresses such as high light stress in poinsettia (*Euphorbia pulcherrima*) plants damage chlorophyll a and b [59]. Thus, drought stress is positively correlated with the degradation of photosynthetic pigments (chlorophyll a and b) in plant leaves. Interestingly in our experiment, after drought stress, wild type plants showed a significant lower contents of chlorophyll a and b than the transgenic lines. After 14 days of drought stress, the wild type and empty vector lines leaves showed an intense decline in chlorophyll a and b contents (Figure 7H,I) and exhibited pale green and shrink phenotypes, which was different than the overexpressed lines (Figure 2B,C). Hence, the retaining of a high level of chlorophyll a and b by the overexpressed lines clearly showed that they had a more resilient ability to tolerate drought stress than the wild type.

Drought stress triggers the production of hydrogen peroxide and malondialdehyde, which causes protein oxidation and damages the cellular components [65]. In plants, a high level of H_2_O_2_ causes redox imbalance (in plant cells, a high production of ROS overwhelms the quenching of free radicals) during a period of progressive dehydration [63]. In addition, a higher electrolyte leakage is accompanied by an accumulation of ROS, and a high level of ROS often results in programmed cell death [66]. A high level of electrolytic leakage shows that the plants are under serve stress [66]. Our drought stress experiment showed lower H_2_O_2_, MDA and electrolytic leakage levels in all transgenic lines than in the wild type plants (Figure 7C–E). In addition, NBT staining showed a lower accumulation of H_2_O_2_ and free radicals (Figure 7B), which showed that the wild type *Arabidopsis* plants were highly sensitive to drought stress, whereas the overexpressed lines were drought-tolerant due to their maintenance of lower H_2_O_2_ and MDA contents and a higher free radical quenching capability (Figure 7C–E).

## 5. Conclusions

From our experiments, we conclude that the overexpression of the citrus *CYTOCHROME P450 75B1* genes significantly accumulates antioxidant flavonoids with increased antioxidant activity and capacity in all transgenic lines; in addition, our gene expression results showed that several genes related to the accumulation of antioxidant flavonoids were up-regulated by 2–12 fold in transgenic lines. Furthermore, 14 days of drought stress conferred more root elongation and more enhanced antioxidant flavonoid and anthocyanin contents in all transgenic lines than in wild type wild plants, and this stress also contributed to drought tolerance. Moreover, after 14 days of drought stress, the transgenic lines showed a significant increment in antioxidant enzymatic activities and possessed a higher total antioxidant potential (free radical scavenging activity) with lower superoxide radicals and reactive oxygen species than the wild type plants. Thus, our phenotypic, gene expression, metabolic, enzymatic and biochemical results demonstrate that overexpression of the *CsCYT75B1* gene from the sweet orange confers drought tolerance in transgenic *Arabidopsis* by triggering the production of antioxidant flavonoids with improved free radical scavenging capabilities.

## Supplementary Materials

The following are available online at https://www.mdpi.com/2076-3921/9/2/161/s1, Figure S1. Heat map and hierarchical cluster analysis (HCA) performed by using the square of peaks of detected metabolites in different citrus germplasm. Figure S2. The gene expression pattern in citrus species at different stages. Figure S3. The gene expression results under drought and high light stress on *Citrus sinensis* leaves. Figure S4. The gene expression data of different genes that were directly or indirectly involved in the biosynthesis of flavonoids. Table S1. The *Arabidopsis thaliana* primers sequence that was used for q-PCR and gene IDs of the transcription factor sand enzymes that involved in the flavonoid and anthocyanin pathway. Table S2. The raw details and modes of the metabolites used in this study. Table S3. The qPCR primer sequence for the citrus varieties used in this study for gene expression analysis. Supplementary File S2. Raw transcriptome data of sweet orange.
