# Supplementary material for: CsCYT75B1, a Citrus CYTOCHROME P450 Gene, Is Involved in Accumulation of Antioxidant Flavonoids and Induces Drought Tolerance in Transgenic Arabidopsis
Source: Antioxidants (Basel). 2020 Feb 17;9(2):161. doi: 10.3390/antiox9020161 (PMC7070963; doi:10.3390/antiox9020161)
Supplement: Supplementary file 1 [file antioxidants-09-00161-s001.zip › antioxidants-706371-supp/Supplementary file 1.docx]

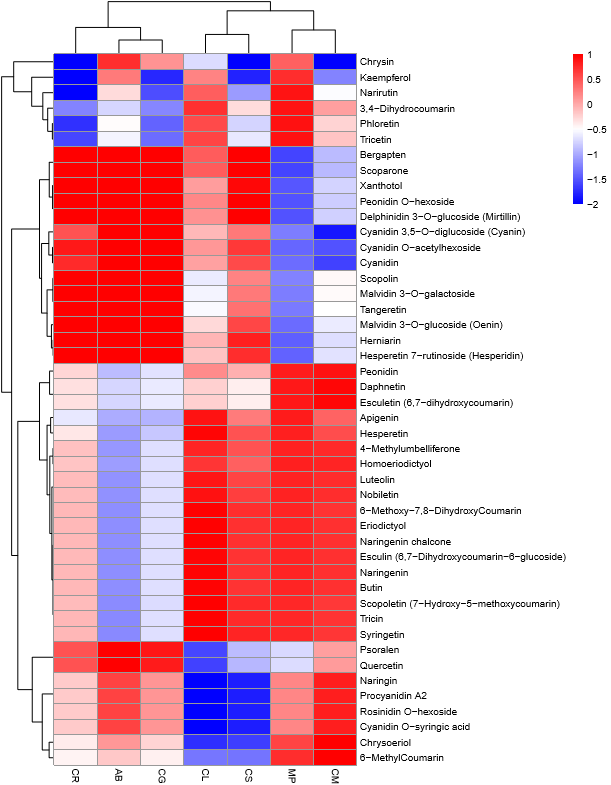


**Figure S1** Heat map and hierarchical cluster analysis (HCA) using the square of peaks of detected metabolites in different citrus germplasm germplasm (AB; *Atalantia buxifolia*, CG; *Citrus grandis*, and CS; *Citrus sinensis*). Column represents varieties and row characterized flavonoids and anthocyanins. Other abbreviations: MP; *Murraya paniculata*, CL; *Citrus latipes*, CM; *Citrus medica*, CR; *Citrus reticulata*.

**Figure S2** Showing the gene expression pattern in citrus species at different stages. SWO: Sweet orange; AB: *Atalantia buxifolia*; CG: *Citrus grandis*.

Then we have grown the sweet orange seedlings and exposed them to different abiotic stress such as drought stress and high light stress. The CsCYP75B1 was high expressed after drought stress (Figure S3). So, we have cloned the CsCYP75B1 gene from sweet orange and overexpressed in *Arabidopsis thaliana* to evaluate its function and possible role in stress.

**Figure S3** Representing the gene expression results under drought and High light stress on *Citrus sinensis* leaves. 14DDS: after 14 days of drought stress; 14 HLS, 14 days of high light stress. Values are mean of three replicates ± SE. Student’s t-test was used to compare control and stressed plants **p* < 0.05. ***P* < 0.01.

**Figure S4** Representing the gene expression data of different gene that directly or indirectly involved in the biosynthsis of flavonoids. WT: wild type, EV: empty vector, OX: overexpressed lines. Students t-test was used to compare CYT-OX and WT at **p* < 0.05 and ***p* < 0.01. Each value are means of three replicates.

**Supplementary Table S1**: Representing the *Arabidopsis thaliana* primers sequence used for q-PCR and gene IDs of transcription factor and enzyme that involved in the flavonoid and anthocyanin pathway.

| **Serial no.** | **Enzyme codes** | **Description** | **Arabidopsis gene ID** | **Primer seq. used for q-PCR (5’ to 3’)** |
| --- | --- | --- | --- | --- |
| 1 | Cs F | CYTOCHROME P450 75B1 | Cs5g11730 | TGGCGGATGCTGAGGAA |
|  | Cs R | CsCYT75B1 |  | GGGCATTCACAACGCACA |
| 2 | CYP F | CYTOCHROME P450 75B1 | AT5G07990 | ATCATCGGAAACCTCCCTCA |
|  | CYP R |  |  | CGAAATTGGCGTCGTGTATT |
| 3 | TT3 F | DFR (Transparent testa 3) | AT5G42800 | CCAAACGCCAAGACGCTAC |
|  | TT3 R |  |  | TTCACTGTCGGCTTTATCACTTC |
| 4 | TT4 F | Transparent testa 4 | AT5G13930 | CGGTCAGGCTCTTTTCAGTG |
|  | TT5 R |  |  | CCTCAAATGTCCGTCTATGGC |
| 5 | TT5 F | Transparent testa 5 | AT3G55120 | TTTGTACCGTCCGTCAAGTCA |
|  | TT5 R |  |  | ATTCTGTTAGCTCCTCCGTAGTTT |
| 6 | TT6 F | Flavanone 3-hydroxylase | AT3G51240 | ACACTGACCCTGGAACCATTAC' |
|  | TT6 R |  |  | ATCAGCATTCTTGAACCTCCC |
| 7 | TT8 F | Athocyanin biosynthesis | AT4G09820 | TCCGAGCAGAACTACCAACG |
|  | TT8 R |  |  | GCCGCCCCTTCAACAAA |
| 8 | TT9 F | Transparent testa 9 | AT3G28430 | CGAGCAGCCGCCTATTTAT |
|  | TT9 R |  |  | TTCAGTTCGGTGCCAGGTT |
| 9 | TT13 F | Transparent testa 13 | AT1G17260 | CAACAGGGTGCCATTACGAA |
|  | TT13 R |  |  | GGTTCCAGTTTTATCACAGCAGA |
| 10 | TT18 F | LDOX | AT4G22880 | ATCGTGGGTTGGTGAATAAGG |
|  | TT18 R |  |  | TTGAGCAAAAGTCCGTGGAG |
| 11 | BAN F | Banyuls | AT1G61720 | ATGCAGAAGCTATCTGGCTCG |
|  | BAN R |  |  | TCCGCAAGAAACAAATGGG |
| 12 | JOX3 F | Jasmonate-induced oxygenase3 | AT3G55970 | ATTATCTCCATTACCAGCCTTCG |
|  | JOX3 R |  |  | CTCGCATAGTTTCACCATTTCC |
| 13 | FLS F | Flavonol synthase | AT5G08640 | CTCCCCGTGAAAAGATTGTTG |
|  | FLS R |  |  | CTTGCGGTAACTGTAATCCTTGA |
| 14 | F | Flavonoids biosynthesis | AT4G12334 | TATGGATATGGTTCTTGGTGGG |
|  | R |  |  | TCCGGTGAGGAACTGTAAGATAA |
| 15 | F6’H1 F | Flavonoids biosynthesis | AT3G13610 | TGATGCTGCTGAGAAATGGG |
|  | F6’H1 R |  |  | TGCTCTGCAAGAGGACTAAAACT |
| 16 | AT-Actin 1 | Internal reference control |  | GGAAGGATCTGTACGGTAAC |
|  | AT-Actin 2 |  |  | TGTGAACGATTCCTGGACCT |

**Table S2** Showing the raw details and mode of metabolites used in this study.

| **Sr No** | **Index** | **Ion mode** | **Molecular Weight (Da)** | **Ionization model** | **KEGG ID** | **Compounds** | **Class** |
| --- | --- | --- | --- | --- | --- | --- | --- |
| 1 | Cit777 | Positive | 580.1792 | [M+H]+ | C09793 | Narirutin | Flavanone |
| 2 | Cit778 | Positive | 580.1792 | [M+H]+ | C09789 | Naringin | Flavanone |
| 3 | Cit781 | Positive | - | [M+H]+ | - | Xanthotol | Coumarins |
| 4 | Cit782 | Positive | 216.0423 | [M+H]+ | C01557 | Bergapten | Coumarins |
| 5 | Cit783 | Positive | 186.0317 | [M+H]+ | C09305 | Psoralen | Coumarins |
| 6 | Cit785 | Positive | 178.0266 | [M+H]+ | C03093 | Daphnetin | Coumarins |
| 7 | Cit786 | Positive | 176.0473 | [M+H]+ | C09268 | Herniarin | Coumarins |
| 8 | Cit788 | Positive | 354.0951 | [M+H]+ | C01527 | Scopolin | Coumarins |
| 9 | Cit326 | Positive | 463.123 | Protonated | - | Peonidin O-hexoside | Anthocyanins |
| 10 | Cit632 | Positive | 330.1 | [M+H]+ | - | Tricin | Flavone |
| 11 | Cit356 | Positive | 477.1 | Protonated | - | Rosinidin O-hexoside | Anthocyanins |
| 12 | Cit463 | Positive | 301.1 | Protonated | C08726 | Peonidin | Anthocyanins |
| 13 | Cit1031 | Negative | 466.1 | [M-H]- | - | Cyanidin O-syringic acid | Anthocyanins |
| 14 | Cit1069 | Negative | 490.1 | [M-H]- | - | Cyanidin O-acetylhexoside | Anthocyanins |
| 15 | Cit1263 | Negative | 286 | [M-H]- | C01514 | Luteolin | Flavone |
| 16 | Cit1291 | Negative | 286.048 | [M-H]- | C05903 | Kaempferol | Flavonol |
| 17 | Cit1265 | Negative | 302.043 | [M-H]- | C00389 | Quercetin | Flavonol |
| 18 | Cit688 | Positive | 254.0579 | [M+H]+ | C10028 | Chrysin | Flavone |
| 19 | Cit1185 | Negative | 580.1792 | [M-H]- | C09789 | Naringenin 7-O-neohesperidoside (Naringin) | Flavanone |
| 20 | Cit631 | Positive | 300.0634 | [M+H]+ | C04293 | Chrysoeriol | Flavone |
| 21 | Cit1287 | Negative | 272.0685 | [M-H]- | C00509 | Naringenin | Flavanone |
| 22 | Cit627 | Positive | 270.0528 | [M+H]+ | C01477 | Apigenin | Flavone |
| 23 | Cit555 | Positive | 176.0473 | [M+H]+ | C03081 | 4-Methylumbelliferone | Coumarins |
| 24 | Cit484 | Positive | 576.1268 | [M+H]+ | C10237 | Procyanidin A2 | Proanthocyanidins |
| 25 | Cit302 | Positive | 493 | Protonated | - | Malvidin 3-O-galactoside | Anthocyanins |
| 26 | Cit309 | Positive | 493.2 | Protonated | C12140 | Malvidin 3-O-glucoside (Oenin) | Anthocyanins |
| 27 | Cit1284 | Negative | 274.084 | [M-H]- | C00774 | Phloretin | Flavanone |
| 28 | Cit225 | Positive | 465.1 | Protonated | C12138 | Delphinidin 3-O-glucoside (Mirtillin) | Anthocyanins |
| 29 | Cit693 | Positive | 402.132 | [M+H]+ | C10112 | Nobiletin | Flavone |
| 30 | Cit709 | Positive | 372.121 | [M+H]+ | C10190 | Tangeretin | Flavone |
| 31 | Cit1259 | Negative | 288.063 | [M-H]- | C05631 | Eriodictyol | Flavanone |
| 32 | Cit222 | Positive | 611 | Protonated | C08639 | Cyanidin 3,5-O-diglucoside (Cyanin) | Anthocyanins |
| 33 | Cit1302 | Negative | 302.079 | [M-H]- | C01709 | Hesperetin | Flavanone |
| 34 | Cit1193 | Negative | 610.19 | [M-H]- | C09755 | Hesperetin 7-rutinoside (Hesperidin) | Flavanone |
| 35 | Cit619 | Positive | 272.069 | [M+H]+ | C06561 | Naringenin chalcone | Flavanone |
| 36 | Cit633 | Positive | 148.052 | [M+H]+ | C02274 | 3,4-Dihydrocoumarin | Coumarins |
| 37 | Cit1194 | Negative | 192.042 | [M-H]- | C01752 | Scopoletin (7-Hydroxy-5-methoxycoumarin) | Coumarins |
| 38 | Cit1231 | Negative | 302.043 | [M-H]- | C10192 | Tricetin | Flavone |
| 39 | Cit652 | Positive | 160.052 | [M+H]+ | - | 6-MethylCoumarin | Coumarins |
| 40 | Cit1299 | Negative | 346.069 | [M-H]- | C11620 | Syringetin | Flavonol |
| 41 | Cit347 | Positive | 178.027 | [M+H]+ | C09263 | Esculetin (6,7-dihydroxycoumarin) | Coumarins |
| 42 | Cit388 | Positive | 208.037 | [M+H]+ | C09265 | 6-Methoxy-7,8-DihydroxyCoumarin | Coumarins |
| 43 | Cit267 | Positive | 340.079 | [M+H]+ | C09264 | Esculin (6,7-Dihydroxycoumarin-6-glucoside) | Coumarins |
| 44 | Cit1294 | Negative | 302.2788 | [M-H]- | C09756 | Homoeriodictyol | Flavanone |
| 45 | Cit1288 | Negative | 272.069 | [M-H]- | C09614 | Butin | Flavone |
| 46 | Cit580 | Positive | 206.058 | [M+H]+ | C09311 | Scoparone | Coumarins |
| 47 | Cit389 | Positive | 287.24 | Protonated | C05905 | Cyanidin | Anthocyanins |

We have selected the 12 key genes from flavonoids biosynthesis pathway and designed their primer and then check their transcriptome data in different citrus varieties (Table S4). We have checked the transcriptome data among different citrus varieties such as *Atalantia buxifolia,* *Citrus grandis* and in *Citrus sinensis* of 12 genes mention in table S4. The CYP75B1 was highly expressed in sweet orange as compared with *Atalantia buxifolia* (Primitive citrus) and *Citrus grandis* (Pumelo) (Figure S2).

**Table S3** Showing qPCR primer sequence for citrus varieties used in this study for gene expression analysis.

| **Serial No.** | **Gene Code** | **Gene annotation** | **Arabidopsis Gene ID** | ***C. sinensis***  **Gene ID** | **Primer Sequence 5’ to 3’** |
| --- | --- | --- | --- | --- | --- |
| **1** | PAL1 | cinnamic acid biosynthetic process | At2g37040 | Cs6g11940 | F: ACATTGCCGGACTGCTCAC  R: AACCCGAAACCCGCTTG |
| **2** | C4H | phenylpropanoid metabolic process | At1g65060 | Cs5g24900 | F: ACTTACTCTTATGCCGAAACGC  R: TGGAAGCTCCCATGAACGA |
| **3** | CHS | flavonoid biosynthetic process | At5g13930 | Cs2g14720 | F: CTTTGTTCGGTGATGGTGCT  R: CCGTCAGAGTCAGGGAGGAT |
| **4** | CHI | flavonoid biosynthetic process | At3g55120 | Cs7g28130 | F: GAGGATTGGAGATTGAAGGGAA  R: ACACTCCTATCGCCGTGAACT |
| **5** | F3’H (CYP75B1) | flavonoid biosynthetic process | At5g07990 | Cs5g11730 | F: TGGCGGATGCTGAGGAA  R: GGGCATTCACAACGCACA |
| **6** | FLS1 | flavonoid biosynthetic process | At5g08640 | Cs1g19280 | F: CTTTCTGCCCTCACCGTTCT  R: CATTCTCGTCTTGTCCTTGCTAA |
| **7** | DFR | anthocyanin-containing compound biosynthetic process | At5g42800 | Cs3g25090 | F: TGGCTATGCTGTTCGTGCTAC  R: TTCCCTCTTCGGCTAAATCG |
| **8** | LDOX/ANS | anthocyanin-containing compound biosynthetic process, | At4g22880 | Cs5g09970 | F: CTTCGGGAAAGATTCAGGGTT'  R: TCGTCGCTAGGCTTCTCAGTT |
| **9** | ANR/BAN | negative regulation of flavonoid biosynthetic process, | At1g61720 | Cs2g07290 | F: CAATCCACCCAAAAGAAGACG  R: ACAGTAGCATGAACAGCATAGC |
| **10** | F3RhaT (UGT78D1) | flavonol biosynthetic process, | At1g30530 | Cs5g24820 | F: TATCAGGGACAAAATAGGCACC  R: TCAGGCAAGTCGGCGACGCGTA |
| **11** | F3GlcT (UGT78D2) | phenylpropanoid metabolic process | At5g17050 | Cs5g24820.1 | F: TATCAGGGACAAAATAGGCACC  R: AACTCCTTCAGGCAAGTCGG |
| **12** | F3AraT (UGT78D3) | Anthocyanidin 3-O-glucosyltransferase 2 | At5g17030 | Cs5g24820 | F: GACAAAATAGGCACCCAAAGTC  R: AACTCCTTCAGGCAAGTCGG |
